# A Case of Radiation-Induced Multifocal Laryngeal Angiosarcoma Presenting as a Diagnostic Dilemma

**DOI:** 10.1155/2012/139310

**Published:** 2012-08-13

**Authors:** Jayme R. Dowdall, Krisha J. Opfermann, Harold Kim, Ho-Sheng Lin

**Affiliations:** ^1^Division of Laryngology, Department of Otology and Laryngology, Harvard Medical School, Massachusetts Eye and Ear Infirmary Boston, MA 02114, USA; ^2^Department of Radiation Oncology, Princess Margaret Hospital, Toronto, ON, Canada M5G 2Mg; ^3^Department of Radiation Oncology, Wayne State University and Karmanos Cancer Institute, Detroit, MI 48201, USA; ^4^Department of Otolaryngology-Head and Neck Surgery, Wayne State University and Karmanos Cancer Institute, Detroit, MI 48201, USA; ^5^Department of Surgery, John D. Dingell VA Medical Center, Detroit, MI 48201, USA

## Abstract

Head and neck sarcomas are relatively rare tumors, with angiosarcomas representing a small subset. Angiosarcoma is a malignant endothelial neoplasm characterized by atypical, multilayered, or solid endothelial proliferation with vasoformative architecture. The global incidence of irradiation-associated sarcoma is estimated as between 0.03% and 0.08%. Here we reported the case of an elderly woman previously treated with radiation more than 20 years ago for an unknown primary of head and neck. This interesting case presented as a diagnostic challenge, and multiple biopsies were required to eventually establish the diagnosis of laryngeal angiosarcoma. We additionally have confirmation from our prior radiation records that the patient did, in fact, receive a substantial dose of radiation to the site previously. To our knowledge, this case represents the first report of a documented radiation-induced multifocal laryngeal angiosarcoma.

## 1. Introduction

Head and neck sarcomas are relatively rare tumors, with angiosarcomas representing a small subset. Angiosarcomas have a higher propensity to affect the elderly and prior studies exist correlating them to radiation exposure. Angiosarcomas tend to originate either from blood vessels or lymphatic vessels. Most head and neck angiosarcomas tend to be observed in the face or scalp. In nonvisible areas of the body, the tumor generally presents as a palpable mass or with symptoms specific to the site. After treatment, the tumor tends to recur locally; however, distant metastases may also occur. The best determinant of overall survival is the ability to completely resect the tumor with a negative and wide margin. While surgery is the main form of treatment, the overall survival for patients with angiosarcoma is low and opportunity of a wide resection margin is rare in the head and neck region. For these reasons, patients with a high-grade lesion and/or positive or close margins may benefit from adjuvant chemoradiation therapy.

We report the case of an elderly woman previously treated with radiation more that 20 years ago for an unknown primary of head and neck who now presented with a fungating supraglottic mass. This interesting case presented as a diagnostic challenge, and multiple biopsies were required to eventually establish the diagnosis of laryngeal angiosarcoma. 

## 2. Case Report

An 83-year-old female was directed to the emergency department by her primary care physician three weeks after falling on her left flank for a CT of her abdomen. This CT showed concern for bile duct dilatation, but as she was asymptomatic, she was discharged home with followup. Several days later an endoscopic retrograde cholangiopancreatography (ERCP) was performed, and a large hypopharyngeal mass was incidentally noted. A biopsy was taken at the time of her GI procedure. The patient was subsequently admitted, and ENT was consulted the following day. Her past medical history includes hypertension, congestive heart failure, stroke, and history of cervical metastasis from unknown primary that was treated with radiation therapy 21 years ago.

This patient was originally diagnosed with a T0N1M0 cancer of unknown primary in 1987. A primary site was not identified on CT of the head, neck, chest, abdomen, and pelvis as well as quadruple endoscopy. Fine-needle aspiration of the neck mass was positive for a poorly differentiated carcinoma. The lesion was 1–1.5 cm in size in the right jugular digastric region just below the angle of the mandible. She received definitive radiation therapy to the head and neck region at Gershenson Radiation Oncology Center, Detroit, MI, from February 11th, 1987 to May 5th, 1987. She received treatment to the neck node and potential sites of primary via left and right lateral opposing ports to 3600 rads by means of Cobalt 60. After this dose, off-cord treatment was delivered to the left and right anterior neck to an additional 1440 rads. Finally, the site of cervical adenopathy was given an additional boost of 900 rads with 10 MV electrons. The lateral supraclavicular field and posterior neck were also treated. On review of the treatment portals and prescribed therapy, the patient received a cumulative dose of 5940 rads to the anterior neck 20 years prior. The anterior neck irradiated included epiglottis and larynx.

On further questioning, patient admitted to one-month history of dysphagia to solids and liquids. She reported a globus-type sensation as well as discomfort when eating. She also had intermittent hot potato voice and coughing episodes. She has had a twenty-pound weight loss and was more easily fatigued. However, she denied any fevers, night sweats, shortness of breath, or otalgia. The patient is a former heavy tobacco user, about a pack a day to a third of a pack a day. She denied any alcohol or drug use. On physical examination, a large and fungating epiglottic mass extending into the base of the tongue was noted on fiberoptic nasopharyngoscopy. Neck examination revealed no obvious lymphadenopathy.

 CT scan of the neck and thorax with IV contrast performed the same day demonstrated a heterogeneous enhancing epiglottic mass which involves the preepiglottic space and extends inferiorly along the mucosal pharyngeal space more on the right involving the laryngeal surface of the aryepiglottic folds. This mass measures 2 × 1.8 × 1.6 cm. The mass extends to involve the false cords and possibly the superior aspect of the right true vocal cord. There is no cartilage invasion, retrocricoid invasion, or subglottic extension. No significant lymphadenopathy. 

The original biopsy at the time of ERCP returned necrotic debris with focal squamous features and cellular atypia. Numerous clusters of bacterial and fungal elements were consistent with candida. The patient was then taken back to the operating room for triple endoscopy, tracheostomy, and biopsies. Direct laryngoscopy revealed a friable, exophytic mass involving the base of tongue, vallecula, and the oral surface of the epiglottis as well as the laryngeal surface of the epiglottis down to the aryepiglottic fold but does not involve the false vocal cord, arytenoid, piriform sinus, or the postcricoid space. The mass was biopsied. Direct bronchoscopy was negative. Direct esophagoscopy revealed a duodenal mass which was biopsied. A PEG tube was placed. The pathology returned predominantly fibrinopurulent exudates and necrotic debris with bacterial and fungal organisms. Because of the negative biopsy, the patient was once again taken to the operating room for biopsy. This was biopsied several times and sent off for frozen section. Frozen section was negative. We then sent off for another set of biopsy specimens. Again this was negative. We sent a third set of biopsy specimens, and this was deferred for permanent. Pathology returned benign squamous mucosa with marked subepithelial inflammation, florid granulation tissue with prominent endothelial proliferation and extensive necrosis. Sections show extensive necrosis with prominent exuberant granulation tissue with spindle-cell proliferation and a chronic inflammatory infiltrate. The spindle cells do not show much cytologic atypia, and no mitotic figures were noted. Some of the spindle cells appear to form vascular channels. The differential diagnosis included florid granulation tissue formation, a poorly differentiated spindle-celled carcinoma or a spindle-cell sarcoma. Immunohistochemical stains were performed and included actin (smooth muscle marker), CD31 (endothelial marker), P63, CAM5.2, and AE1/AE3 epithelial markers. Sections showed the spindle cells to stain strongly with the CD31, while the other stains were essentially negative in the spindle cell area. This is consistent with a florid endothelial proliferation or granulation tissue formation. No unequivocal malignancy is seen in this specimen.

**Figure 1:**
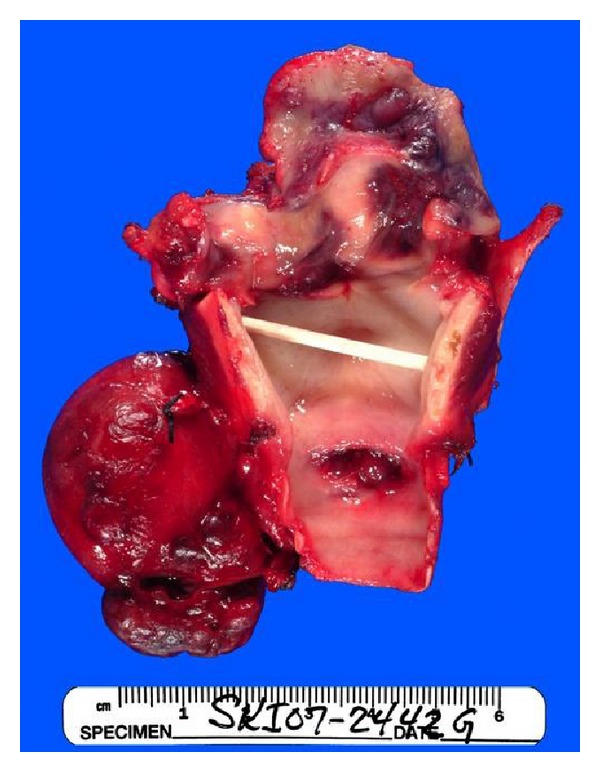


**Figure 2:**
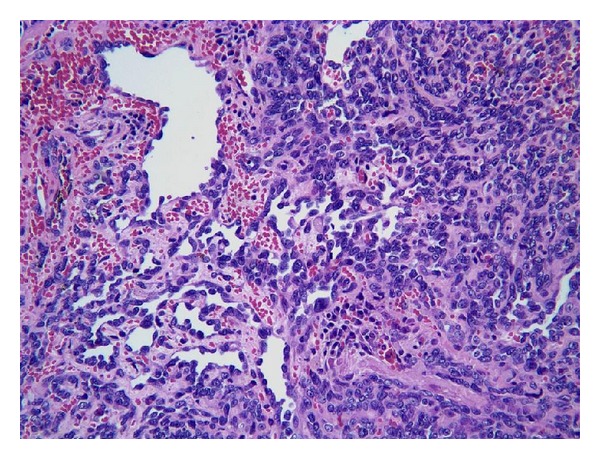


**Figure 3:**
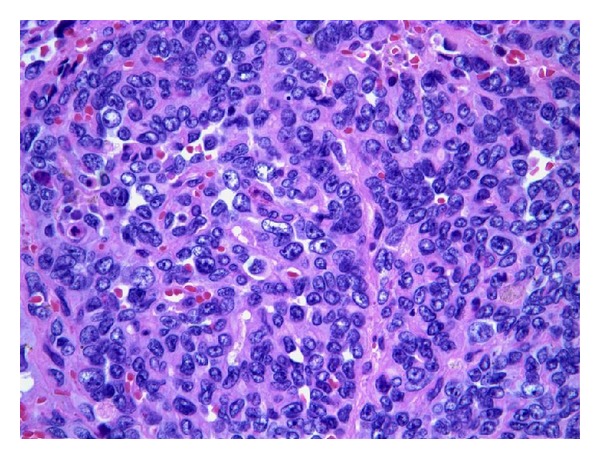


After three negative and inconclusive biopsies, patient's case was discussed in the Multidisciplinary Head and Neck Tumor Board, and a recommendation for a larger and more aggressive biopsy was recommended. Infectious disease has also been consulted regarding this patient, and they requested tissue for cultures. After extensive discussion with the patient and her family, patient was taken back to OR for extensive debulking of the epiglottic mass. The final pathologic diagnosis was angiosarcoma. The biopsy shows a highly cellular neoplasm with necrosis. Tumor cells are positive for CD31, CD34, and negative for Factor VIII, cytokeratin AE1/AE3 and CK903. The morphology and immunoprofile are diagnostic of epithelioid angiosarcoma. Outside consultation from the University of Michigan ultimately agreed with the above diagnosis. The patient then underwent a total laryngectomy and cricopharyngeal myotomy with tracheoesophageal puncture. During the surgery, 2 primary foci involving the epiglottis and base of the tongue were noted (Figure 1). The final pathology confirmed the presence of multifocality with a 2.2 cm lesion in the epiglottis and a 1.5 cm lesion in the base of tongue. All margins were negative, and the pathology showed high-grade epitheliod angiosarcoma of the epiglottis (Figures 2 and 3). Unfortunately, the patient developed several complications, including fistula, syncope, seizure, and pneumonia, following the surgery, and discussion of adjuvant chemoradiation therapy was delayed. Patient was doing well until approximately 10 months after the surgery when she was noted to have a purplish lesion in the right tonsil area. CT scan of neck and chest was negative. Examination under anesthesia revealed 2 foci of lesion, one in the right tonsil and one in the left pharynx. Biopsy showed angiosarcoma. Patient was taken back to the OR for right radical tonsillectomy and L partial pharyngectomy. Patient did well postoperatively and was started on adjuvant chemotherapy consisting of taxotere. Approximately 6 months after the last surgery, patient was again noted to have purplish lesion in the left base of tongue region. She was experiencing significant side effects from the chemotherapy, and family decided to put her in hospice. She died 2 months later. 

## 3. Discussion

Primary laryngeal sarcomas have been determined to comprise only 0.32%, 0.55%, and 0.87% of all laryngeal malignancies based on previous studies [2]. Of these, chondrosarcoma is the most common, followed by fibrosarcoma. Angiosarcoma constitutes a smaller subset of the malignant sarcomas. Angiosarcoma of the larynx is a rare malignant tumor, accounting for less than 1% of all malignant tumors of the larynx [5]. Of the laryngeal angiosarcomas analyzed by Miuraet al. most arose from the supraglottis (64.3%), often with epiglottic invasion, followed by the glottis (28.6%), and subglottis (7.1%) [2, 4].

Angiosarcoma is a malignant endothelial neoplasm characterized by atypical, multilayered, or solid endothelial proliferation with vasoformative architecture [2]. The global incidence of irradiation-associated sarcoma is estimated as between 0.03% and 0.08% [6]. Eleven cases of angiosarcoma of the head and neck were reviewed the mean age was determined to be 64 years; surgery was the primary method of treatment; 2-year survival rate was 50%; 5-year survival rate was 22%. Regional metastases were seen in 18%. In this study, the tumors were found to be poorly, circumscribed and tended to spread horizontally within the dermis for considerable distances [7]. Another study of primary laryngeal sarcomas found the mean age to be 62.2 years, a predominance for male patients: 9 : 5 with the main symptoms being hoarseness followed by hemoptysis. The symptoms were present for a mean of 6.3 months on diagnosis [2]. Angiosarcomas account for only 1% of all soft tissue sarcomas, 60% of which are cutaneous. The occurrence of an angiosarcoma in the oral cavity is unusual. About 4% of angiosarcomas have been found in the paranasal sinuses, oral cavity, or pharynx [8]. Although angiosarcoma can occur in any organ, it typically presents: (1) in the face or scalp among elderly, (2) with chronic lymphedema, (3) after radiotherapy, or (4) as a hepatic primary [9]. The least frequent is one which develops following previous radiation therapy [8]. Some cases are believed to be radiation-induced. Other possible causative factors include: trauma, lymphoedema, foreign bodies, vinyl chloride and, arsenic [2].

Only 13 case reports of primary laryngeal angiosarcoma have been reported in the English literature to date Tables 1 and 2. Only 2 of those documented as possibly radiation-induced [2]. Also, only one of the laryngeal angiosarcomas is specifically determined to be of origin in the epiglottis; however, in this case, the patient had not received prior radiation [10]. In the case report not only we present our patient definitively that is diagnosed with an angiosarcoma of the epiglottis, but also we have confirmation from our prior radiation records that the patient did, in fact, receive a substantial dose of radiation to the site previously. In order to attribute to prior radiation treatment as the causative factor the following classification has to be met: (i) the site of origin must be within the field of previous radiation; (ii) the patient should have received a significant amount of radiation therapy (greater than 2,500 rads); (iii) an interval of at least 3–5 years must elapse between the time of irradiation and the development of the sarcoma; (iv) the second primary cancer need is histologically different from the primary neoplasm (Table 3) [2, 6]. In the case of our patient: the epiglottis was included in the site of the prior radiation field encompassing the boost, the patient received 5,940 rads, an interval of 20 years had elapsed between her last cancer and the development of the angiosarcoma, and the primary cancer 20 years ago had been histologically identified as squamous cell carcinoma.

Radiation therapy has been well documented as a causative factor in the formation of basal-cell carcinoma and squamous cell carcinoma, but only rarely angiosarcoma [8]. It is viewed as a rare, but possible late complication of radiotherapy in patients who have received a significant amount of irradiation, usually between 25 and 60 Gy. There is usually a latency period of at least 10 years or longer. In a review of postradiation soft-tissue sarcomas (53 cases, one an angiosarcoma), the tumors were correlated with the characteristics of the radiation. No difference was noted between the patients treated with megavoltage radiation and with orthovoltage radiation in the type of sarcoma, location, or survival. The orthovoltage group had received a lower mean radiation dose (4446 rads), but the megavoltage group was associated with a shorter latency period [3].

Immunohistochemical and histologic features, are required for identification. Histologically, in angiosarcomas the most common type is epitheliod angiosarcoma [11]. Frequently, the tumors will have anastomosing vascular channels lined by remarkably atypical mitotic figures, and hemorrhage [4]. Immunohistochemical staining with factor VIII-related antigen of a plasma glycoprotein synthesized by endothelial cells [8]. Angiosarcoma can be confirmed immunohistologically by using endothelial markers such as factor VIII-related antigen, UEA I, CD31, and CD 34. However, these markers are not labeled for all angiosarcomas because endothelial differentiation markers are expressed differently from case to case and even within the same tumor. Of the markers, factor VIII-related antigen immunostaining was generally the most focal. Immunostaining of cytokeratin and vimentin, at least, is mandatory to ascertain the diagnosis if secondary sarcoma is suspected [2]. In the cases studied by Meis-Kindblom and Kindblom, all 42 cases stained at least focally for factor VIII-related antigen, and nearly all stained strongly for vimentin, which accentuated the endothelial cells and vessel lumen formation. CD34 antigen was detected in 74% of cases, BNH9 in 72%, and cytokeratins in 35% [11].

The overall prognosis of angiosarcoma is poor. They spread by both the lymph system and via blood vessels [12]. Regarding tumor size, the larger the tumor, the worse the prognosis. Pedunculated and small-size tumors show a better outcome. Poor prognosis is also related to an older patient age, retroperitoneal location, and high MIB-1 labeling index ≥10% of tumor cell population [2, 11]. Lydiatt et al. treated 18 patients from 1978 to 1992 with 16 scalp angiosarcomas and 2 in the oropharynx. Primary surgery was utilized in 9 of the patients, 1 received adjuvant chemotherapy, and 2 received adjuvant radiotherapy. The overall 5-year survival was 33%, but only 20% of the patients were disease free. Once again, size of the tumor was an important predictor, patients with lesion >10 cm died of disease, compared with 67% with a lesion <10 cm. Four of six patients treated with wide local excision for lesions <10 cm survived 5 years [13]. Although laryngeal angiosarcoma is extremely rare and as of 2003 there are 14 documented cases in the literature [2], there may be more specific diagnostic and therapeutic challenges associated with documented radiation angiosarcoma as compared to cases that were not previously irradiated (Table 1). For example, Muira et al. [2] report in 2003 the second case of radiation-induced laryngeal angiosarcoma in a patient who had previously been treated with radiation for tuberculous lymphadenopathy at age 28 and chemo and radiation for supraglottic squamous cell carcinoma at age 60. The patient subsequently had a total laryngectomy for what was thought to be recurrent SCCA based on the biopsy results. Final pathology returned positive for angiosarcoma. This patient also recurred with skin, breast, and muscle metastasis after one year and 10 months each of which was treated with excision and chemotherapy. As of the date of the report the patient was stable for 3 years and six months after total laryngectomy [2].

Thomas [1] reports a case of a 61- year-old male who underwent radiation for SCCA of the vocal folds treated with 70 Gy. Eleven years later the patient developed hoarseness, exam revealed a laryngeal mass which biopsies demonstrated dysplasia with dilation of capillaries and lymphatics without infiltrative growth. The patient underwent laryngopharyngectomy, and final pathology was angiosarcoma. The patient had local recurrence and expired after 13 months. Thomas and Muira both underscore the both the diagnostic dilemma and the multifocality of disease than can occur in the subset of previously irradiated angiosarcomas of the larynx. Many of the rare published reports of angiosarcoma did not fit diagnostic criteria with immunostaining, and there may be a possibility for over diagnosis [2]. Of note, a prior literature review of cutaneous angiosarcoma of the head and neck, three of the ten cases initially diagnosed as angiosarcoma were changed to eosinophilic granuloma, SCCA, and hemangioendothelioma underscoring the diagnostic difficulty [12].

In addition to the pathologic diagnostic dilemma associated with this condition, cases must also fulfill criteria for radiation-induced sarcoma. Although several authors report prior radiation in association with cases of angiosarcoma of the larynx (Table 2), specific criteria must be met to diagnose a radiation-induced sarcoma. Our current case meets all of these criteria.

## Figures and Tables

**Table 1:** Cases that fulfill diagnostic criteria for radiation-induced angiosarcoma of the larynx.

Author	Initial primary	RT	Latency	Pre-op biopsy	Final pathology	Recurrence/multifocality	Survival
Thomas 1979 [1]	Vocal cord SCCA	70 Gy	11 years	Dysplasia with dilation of capillaries and lymphatics, no infiltrative growth	Supraglottic Angiosarcoma	Yes, local recurrence, no multifocal lesions, had pharyngocutaneous fistula	13 months s/p partial pharyngolaryngectomy, final pathology angiosarcoma

Miura et al. 2003 [2]	Supraglottis SCCA, cervical TB	68.4 Gy; unknown 42 years	10 years	Initial biopsy interpreted as SCCA	Supraglottic Angiosarcoma	Skin, breast and muscle	3 years and six months after laryngectomy followed by excision and chemo for metastatic lesions

Current case	Unknown primary SCCA	59.4 Gy	20 years	Initial biopsy inconclusive × 3, 4th biopsy angiosarcoma	Supraglottic Angiosarcoma	Yes, 2 foci epiglottis and base of tongue, yeast recurrence	2 years after narrow-field laryngectomy

**Table 2:** Radiation-associated angiosarcoma of the larynx.

Author	Angiosarcoma site	Initial primary	RT	Latency
Laskin et al. 1988 [3, #212]	Larynx	Larynx SCCA	68 Gy	3 years
Loos et al. 2001 [4, #11]	Supraglottis	Breast Ca, cervical lymph nodes	66 Gy	7 months
Loos et al. 2001 [4, #11]	Supraglottis	Unknown primary	Unknown dose	10 years

**Table 3:** Criteria for radiation-induced malignancy.

(1) The site of origin must be within the field of previous radiation	
(2) The patient should have received a significant amount of radiation therapy (greater than 2,500 rads)	
(3) An interval of at least 3–5 years must elapse between the time of irradiation and the development of the sarcoma	
(4) The second primary cancer needs to be histologically different from the primary neoplasm	
